# Male partner roles in cervical cancer transmission and prevention in central Kenya: A quantitative approach

**DOI:** 10.4102/hsag.v30i0.2858

**Published:** 2025-05-09

**Authors:** John H. Mwangi, Gloria N. Mtshali, Pretty Mbeje

**Affiliations:** 1 School of Nursing and Public Health, College of Health Sciences, University of KwaZulu-Natal, Durban, South Africa

**Keywords:** male partner’s involvement, cervical cancer, transmission, prevention, Central Kenya

## Abstract

**Background:**

Cervical cancer continues to be fatal on a global scale, particularly impacting women during their prime years of productivity. Male partners are an important factor in transmission, prevention and control of cervical cancer.

**Aim:**

The study aimed at identifying couples’ perception on the roles, patterns and factors associated with male partner involvement in transmission, prevention and control of cervical cancer.

**Setting:**

The study was conducted in three public county hospital clinics in Central Kenya.

**Methods:**

The study used cross-sectional descriptive quantitative design where a total number of 358 couples participated in the study.

**Results:**

Some of the factors that couples perceived to affect male partner’s participation were individual characteristics such as marital status (*p* = 0.017), occupation (*p* < 0.000), income (*p* = 0.04), place of residence (*p* = 0.000), health facility factors including friendly affordable services (*p* = 0.025), places for health service delivery (*p* < 0.001) and male friendly services (*p* = 0.000). The community structures and leadership patterns influenced male participation in cervical cancer prevention and control. Male partners were involved with financial and logistic support, moral support, human papillomavirus transmission and vaccination, traditional and cultural practices, health education and health care provision.

**Conclusion:**

Couples felt that male partners had an important part to play in cervical cancer transmission, prevention and control, which was influenced by personal constructs, health care systems and family and/or community factors.

**Contribution:**

Policy makers can incorporate the study findings in policy development and implementation addressing the knowledge gaps, misconceptions and potential barriers that men may face in cervical cancer transmission, prevention and control.

## Introduction

According to the World Health Organization (WHO), Kenya had an incidence rate of 33 per 100 000 women for cervical cancer, with 22 per 100, 000 succumbing to the disease in 2020 (WHO 2020). Exploring strategies to augment knowledge about human papillomavirus (HPV) and cancer screening and fostering trust in the healthcare system among male spouses or partners is essential, particularly with the aim of promoting cervical cancer awareness (Read et al. 2020).

Cervical cancer stands out as one of the diseases that reveal disparities on a large scale. In middle-income countries, its occurrence is twice as high and mortality rates are three times higher compared to high-income countries (Saleh et al. 2016). Unfortunately, the majority of these women go undetected and are unable to obtain life-prolonging treatments that could improve their quality of life. Cervical cancer is the second most common malignancy in regions with a lower Human Development Index (HDI), behind breast cancer. Surprisingly, it is the most often detected cancer in 23 countries and the most common cause of death in 36 nations, primarily in sub-Saharan Africa and Southeast Asia (Sung et al. 2021).

Global cancer statistics underscore the highest incidence and mortality rates for cervical cancer in Africa, particularly notable in Southern Africa, Eastern Africa and Western Africa (Bray et al. 2018). In response to this urgent situation, the WHO launched a Global Strategy with the goal of intensifying preventive, screening and treatment measures to eliminate cervical cancer as a public health concern during the 21st century (Arbyn et al. 2020). This initiative specifically addressed the pivotal issue of male engagement in the sexual and reproductive health (SRH) of their partners. However, minimal progress has been observed in enhancing male involvement in reproductive issues post-initiative (Aborigo et al. 2018).

Studies demonstrate a favourable connection between men’s proactive engagement in reproductive healthcare and enhanced outcomes for maternal and child health (MCH) (Bishwajit et al. 2017). To bridge this disparity, there is an urgent requirement for enduring community health education and promotion strategies that inspire and foster male involvement across various facets of reproductive healthcare, encompassing cervical cancer screening and treatment (Bola-Oyebamiji et al. 2024).

In response to the Kenya’s cervical cancer incidence and mortality, and in an effort to improve cervical cancer prevention, the Ministry of Health (MOH) in Kenya released the National Cervical Cancer Prevention Program (NCCPP) Strategic Plan (2012–2015) (MOH 2015). One objective, among several key objectives and strategies, prioritised providing high-quality services and outlined associated strategies, including reducing the incidence and prevalence of cervical cancer and providing cervical cancer screening. To meet these objectives, the plan specified that a key programme output was to create awareness among relevant authorities and personnel’s including eligible women and their partners on the need to prevent cervical cancer through utilisation of the availed preventive and treatment services (MOH 2015).

Cervical cancer disproportionately affects individuals in the younger age group (under 25 years) because of early sexual activity, multiple sexual partners and a history of sexually transmitted diseases, primarily associated with HPV (Bouassa et al. 2017). In Kenya, HPV testing is recommended as the primary screening method for women aged 30 years and above. In situations where HPV testing is unavailable or poses a risk of loss to follow-up, visual inspection with acetic acid (VIA) or visual inspection with Lugol’s iodine (VIA or VILI) is suggested as the primary screening method (Saleh et al. 2016).

Male partners or significant others can influence women’s decisions regarding the uptake of cervical cancer screening and treatment services, impacting logistical, educational and psychosocial factors. These factors encompass both positive facilitators, such as emotional support, encouragement and financial assistance, as well as potential negative impacts such as stigmatisation, isolation or outright prohibition of access to care (Sharma, Kc & Khatri 2018). Historically, SRH initiatives have predominantly centred around women. While strategies integrating gender issues remain crucial, recognising the direct involvement of male partners in SRH discussions has gained prominence as a key determinant of acceptance and care uptake (Adewumi et al. 2019). Research indicates that both women and men express interest in shared decision-making in reproductive matters, with the influence of partners linked to women’s adherence to reproductive health issues (Aborigo et al. 2018; Adewumi et al. 2019).

Within African cultures, men continue to hold a dominant role in family structures. This social framework, emphasising male leadership, can be utilised in health promotion interventions designed to enhance women’s engagement in cervical cancer screening (Adegboyega et al. 2019). A study done in Nyeri (Central Kenya) on factors influencing utilisation of cervical cancer screening services recommended the MOH and other agencies including individuals of goodwill to collaborate in designing and implementing awareness campaigns on cervical cancer screening that target both men and women in the community. It also advocated male partner support in cervical cancer prevention and control processes (Wangeci & Macharia 2018).

Kenya cancer policy 2019–2030 has addressed many areas in regard to cancer prevention and management (MOH 2020); however, nothing is mentioned on partners support nor male input in prevention and controlling cervical cancer.

While the introduction of the HPV vaccine in certain areas of Kenya may have increased community awareness of cervical cancer, insufficient levels of knowledge and awareness could contribute to suboptimal screening rates. Although various barriers at the community, patient and provider levels may exist, a detailed exploration of these factors is lacking (Buchanan Lunsford et al. 2017).

Previous studies have investigated factors influencing the low uptake of available cervical cancer prevention services. However, limited attention has been given to the participation or involvement of male partners. Furthermore, there are no published studies that have examined the input and impact of male partner participation on the utilisation of provided cervical cancer screening and treatment services. Research on the extent of male partner influence and the potential advantages and disadvantages of male involvement in cervical cancer prevention is scarce, including considerations of acceptability and desirability (Adewumi et al. 2019). Despite the critical role male partners can play in alleviating the burden of cervical cancer, there is minimal information on their involvement in the screening and treatment process (Binka et al. 2019).

A significant cause-and-effect association is found between cancer of the cervix and repeated HPV infections over time (Dillner & Brown 2004). Human papillomavirus can be transmitted from an infected man to an uninfected woman through sexual means (Denny et al. 2010). After transmission of oncogenic HPV, cervical cancer develops slowly, and signs and symptoms may take many years before they appear (Dillner & Brown 2004). This means that even though women carry the burden of the disease, male sexual partners play a role in the transmission of the virus to their female partners (Moodley & De Vries 2016).

Spousal support in health matters has a positive impact on health promotion and the mitigation of ill health, as indicated by Adegboyega et al. (2019). Identifying the roles, patterns and factors associated with male partner involvement in the cervical cancer transmission and uptake of screening and treatment services will enhance the implementation of programmes at the facility, county and country levels in Kenya.

## Research methods and design

### Study design

The study employed a cross-sectional descriptive quantitative design to find out the perception of couples on roles, patterns and factors associated with male partner involvement in transmission, prevention and control of cervical cancer.

### Study setting

This study was done in three county referral hospitals MCH clinics in central Kenya, namely Murang’a, Nyeri and Kirinyaga. Based on data from Hospital Registries, Central Kenya and Nairobi have the highest burden of cervical cancer in Kenya.

### Population and sampling

Fisher’s formula was used to get a sample size of 358 couples from the selected county hospital MCH clinics. This sample was allocated on pro-rata basis. A sample population of 156 couples, 112 couples and 90 couples was selected from Murang’a, Nyeri and Kirinyaga, respectively. Systematic random sampling was used to get the samples in the selected clinics, where if they met the selection criteria, they were included in the study. Couples were given health education in the MCH clinic where the researcher or the assistants introduced themselves and explained about the study including the process and benefits. Every third couple within the sitting arrangement was included in the sample provided they met the criteria and were willing to participate.

### Inclusion criteria

Women and their male partners who attended MCH clinics in the study area and provided written consent to participate in the study were included in the study.

### Exclusion criteria

Participants in the study area who declined to provide consent for participation in the study were excluded.

### Research instruments

The data collection instruments included structured questionnaire developed and designed by the researchers in line with the research aim and objectives. There were three components to the questionnaire: a part on demographic details and individual characteristics, section on health systems and the section on family and/or community support.

### Validity and reliability of the research instrument

The tools underwent pre-testing in an adjacent area (Nyandarua County) with participants sharing characteristics similar to those in the main study area to ensure internal validity. The researcher employed three distinct strategies for a rigorous pre-test assessment of the study tools: behaviour coding, individual briefings and specific methodologies (cognitive interviewing and readability testing). Results from the pre-test demonstrated strong internal consistency and reliability of the instruments, evidenced by a Cronbach’s alpha of 0.8. Feedback obtained from the pre-test phase guided subsequent refinements and the finalisation of the study instrument.

### Data collection

Data were collected over 9 weeks (from 08 April 2024 to 07 June 2024) at the MCH clinics of three selected county hospitals. The researcher, along with assistants, distributed structured questionnaires to the study participants, providing assistance to those with reading difficulties. The questionnaire was available in both English and Swahili. Before collecting data and obtaining informed consent, the researcher provided a brief explanation of the study and its purpose to the participants.

### Data analysis and interpretation

The data were analysed using IBM SPSS version 27 (IBM Corporation, Armonk, New York, US). Descriptive statistics were utilised to present demographic variables, with the results expressed as mean and standard deviation. Chi square tests were done to check the association between independent and dependent variables. Regression analysis was also done to test for the estimation of relationships between some of the variables.

### Ethical considerations

The initial step involved obtaining research approval from the University of KwaZulu-Natal’s Biomedical Research Ethics Committee (BREC) (reference no.: BREC/00006580/2023). The respondents participated in the study voluntarily. Data collection commenced after informed consent was obtained from the study participants, who were informed of their right to withdraw at any time. Privacy and confidentiality were strictly maintained, ensuring that data could not be traced back to the participants. Robust security measures to prevent unauthorised access, data breaches and accidental data loss that included strong passwords, encryption and secure storage systems were employed.

## Results

A total number of 358 couples volunteered to participate in the study, and all their responses were subjected to quantitative data analysis.

### Socio-demographic and economic characteristics of the study participants

The majority of males were between the ages of 32 years and 43 years (Table 1) accounting for 51.7% (*n* = 185), while the majority of females were between the ages of 28 years and 38 years, representing 47.2% (*n* = 169). Most couples were customarily married, comprising 54.5% (*n* = 195). Regarding education, the majority of males (48%, *n* = 172) and females (54.2%, *n* = 194) had completed secondary school. Self-employment was common among 41.9% (*n* = 150) of males and 39.7% (*n* = 142) of females. Most couples resided in rural areas, with 48.3% (*n* = 173) of males and 51.4% (*n* = 184) of females living in these settings. The primary income earner was predominantly male (57.5%, *n* = 206).

**TABLE 1:** Socio-demographic and economic characteristics of the respondents.

Attributes	Characteristics	Number	%
Age (years) (males)	20–31	115	32.1
	32–43	185	51.7
	44–55	54	15.1
	56–67	4	1.1
Age(years) (females)	17–27	125	34.9
	28–38	169	47.2
	39–49	59	16.5
	50–60	5	1.4
Marital status	Cohabiting	115	32.1
	Customarily marriage	195	54.5
	Civil marriage	46	12.8
	Others	2	0.6
Level of education (males)	Primary	47	13.1
	Secondary	172	48.0
	Tertiary	137	38.3
	Others	2	0.6
Level of education (Females)	Primary	68	19.0
	Secondary	194	54.2
	Tertiary	96	26.8
Occupation (Males)	Formal employment	121	33.8
	Casual labourer	49	13.7
	Self employed	150	41.9
	Unemployed	36	10.0
	In school	2	0.6
Occupation (Females)	Formal employment	68	19.0
	Casual labourer	39	10.9
	Self employed	142	39.7
	Unemployed	87	24.3
	In school	22	6.1
Place of residence (Male)	Rural	173	48.3
	Urban	125	34.9
	Peri urban	60	16.8
Place of residence (Female)	Rural	184	51.4
	Urban	108	30.2
	Peri urban	66	18.4
Main income earner	Female	89	24.9
	Male	206	57.5
	Both	59	16.5
	Missing	4	1.1

### Health care system attributes

As illustrated in Table 2, to ensure the sustainability of male involvement in cervical cancer screening services, the majority of couples (72.6%, *n* = 260) preferred that clinics be open on weekdays, while 26.5% (*n* = 95) favoured weekend availability. Less than 1.0% (*n* = 3) were indifferent to either option. Majority of the couple participants wanted the waiting time to be less than 1 h (83.2%, *n* = 298) while 16.8% (*n* = 60) were not bothered by waiting for an hour or more. Majority of the couples preferred the hospital to be less than one km from their homesteads (68.2%, *n* = 244), 25.7% (*n* = 92) wanted it to be at least one km while 6.1% (*n* = 22) were okay even when the hospital providing cancer of the cervix screening services was more than one km. Majority of the couples preferred the hospital charges to be less than 100 Kenyan shillings (Ksh) (51.4%, *n* = 184), 36.0% (*n* = 129) wanted it to be at least 100 Ksh and 5.9% (*n* = 21) were okay with more than 100 Ksh. Noteworthy is that only 6.7% (*n* = 24) wanted the screening services to be free.

**TABLE 2:** Strategies for improving male partner participation in cervical cancer prevention and control services (*N* = 358).

Attributes	Category	Number	%
Convenient working day	Weekdays	260	72.63
	Weekends	95	26.53
	Others	3	0.84
Waiting time	< 1 h	298	83.20
	1 h	60	16.80
Distance to the health facility	< 1 km	244	68.20
	1 km	92	25.70
	> 1 km	22	6.10
Friendly/affordable cost	< 100 Ksh	184	51.40
	100 Ksh	129	36.00
	> 100 Ksh	21	5.90
	Free	24	6.70

Ksh, Kenyan Shillings.

### Health care provider’s characteristics and type of male-friendly health services offered

Couples preferred health workers who were welcoming (28.5%, *n* = 102), polite (28.2%, *n* = 101), non-judgemental (22.1%, *n* = 79) and confidential (20.9%, *n* = 75). The type of male-friendly services the couples preferred during the cervical cancer prevention and control health services included more male health care providers (31.6%, *n* = 113), male partner-oriented services such as prostate cancer screening (25.4%, *n* = 91), health education focussing on male partners (23.2%, *n* = 83). A significant portion of respondents emphasised the importance of gender-sensitive seating arrangements (10.9%, *n* = 39) and treatment of sexually transmitted infections (8.9%, *n* = 32) to enhance male involvement in cervical cancer screening services. Half of the couples (50.0%, *n* = 179) preferred that male-friendly services be offered through community outreach setups. In contrast, 43.6% (*n* = 156) favoured maintaining these services within clinic settings, while a smaller group (6.2%, *n* = 22) supported the idea of providing services in institutional environments such as schools.

### Family and community support attributes

The majority of couples felt that male partners could be more actively involved in cervical cancer prevention and control through several approaches. Key strategies as shown in Table 3 included offering moral encouragement for attending checkups (26.8%, *n* = 96), providing financial resources (25.1%, *n* = 90) and accompanying their female partners to the clinic (19.0%, *n* = 68). Other important methods were giving advice (12.6%, *n* = 45), being less judgemental (12.6%, *n* = 45) and avoiding discrimination and stigmatisation (3.6%, *n* = 13).

**TABLE 3:** Male partner involvement in cervical cancer screening.

Responses	Frequency	Percentage	Valid percent	Cumulative percent
Provide resources	90	25.1	25.1	25.1
Give advice	45	12.6	12.6	37.7
Be less judgemental	45	12.6	12.6	50.3
Avoid discrimination/stigmatisation	13	3.6	3.6	53.9
Encourage me to go for checkups	96	26.8	26.8	80.7
Accompany me to the hospital	68	19.0	19.0	99.7
Others	1	0.3	0.3	100.0

Total	358	100.0	100.0	-

Note: The question asked was *‘How can your partner be involved in cervical cancer screening?’.*

### Knowledge of human papillomavirus transmission

Majority of the couples 73.7% (*n* = 264) were not aware that HPV can be transmitted sexually, while 26.3% (*n* = 94) were aware about this.

### Taking children for human papillomavirus vaccination

Majority (76.5%, *n* = 274) of the couples were willing to have their children receive HPV vaccines, whereas 23.5% (*n* = 84) were not.

Some of the reasons for not using the HPV vaccination services were unawareness of existence of such services (18.4%, *n* = 66), the lack of trust of the HPV vaccines (3.9%, *n* = 14) and unawareness of where the vaccination services are offered (1.1%, *n* = 4).

Some of the community members the couples felt could assist in encouraging male partners to support their women to screen for cervical cancer included: community health workers (46.9%, *n* = 168), community leaders (33.5%, *n* = 120), peers or friends (11.7%, *n* = 42) and teachers (2.5%, *n* = 9).

Women were asked independently and confidentially about their intention to involve their male partners during the cervical cancer screening processes. Majority (81.3%, *n* = 291) were willing to involve their men while 18.7% (*n* = 67) were not willing.

The reasons for involving their male partner included financial support (58.8%, *n* = 210), moral support (27.1%, *n* = 97), to understand the process (7.2%, *n* = 28), out of love for them (3.8%, *n* = 13), it is their marital duty (2.4%, *n* = 8), while 0.8% (*n* = 3) did not know why.

Some of the reasons, as depicted in Table 4, for not involving their male partner in the cervical cancer screening processes included being busy, that is not being available (6.7%, *n* = 24), not concerned or supportive about the screening services (4.7%, *n* = 17), women liking their privacy and confidentiality (3.1%, *n* = 11), their men being judgemental (2.2%, *n* = 8) and their men being shy (1.4%, *n* = 5) while 0.6% (*n* = 2) did not have a reason.

**TABLE 4:** Reasons for not involving male partner in cervical cancer screening.

Reasons	Frequency	Percentage	Cumulative percent
Not available (busy)	24	6.7	6.7
Not concerned (not supportive)	17	4.7	11.4
Privacy and confidentiality	11	3.1	14.5
Will be judgemental	8	2.2	16.7
He is shy	5	1.4	18.1
Others	2	0.6	18.7

Total	67	18.7	-

### Evaluation of the association between socio-demographic characteristic and health care system for male partner participation

#### Convenient working days

As illustrated in Table 5, the relationship between convenient working days and socio-demographic and economic characteristics was statistically significant at a significance level of *p* ≤ 0.05. Specifically, the *p*-values were as follows: *p* = 0.003 for males’ age, *p* = 0.0226 for females’ age, *p* = 0.038 for marital status, *p* = 0.0004 for males’ level of education, *p* = 0.000 for females’ level of education, *p* = 0.0056 for males’ occupation, *p* = 0.003 for females’ occupation and *p* = 0.000 for both males’ and females’ place of residence.

**TABLE 5:** Socio-demographic characteristics and convenient days for male partner participation in cervical cancer screening.

Variables		Convenient working days (*n*)		*χ*^2^ value	*df* value	*p*-value
Attribute	Characteristics	Week days	Weekends			
Age (years)(males)	20–31	74	41	18.581	3	*p* = 0.0003
	32–43	151	31			
	44–55	33	21			
	56–67	2	2			
Age (years) (females)	17–27	79	43	9.568	3	*p* = 0.0226
	28–38	136	33			
	39–49	42	17			
	50–60	3	2			
Marital status	Cohabiting	78	37	8.3858	3	*p* = 0.038
	Customarily marriage	153	42			
	Civil Marriage	28	18			
	Others	1	1			
Level of education (Males)	Primary	45	2	18.038	3	*p* = 0.0004
	Secondary	126	46			
	Tertiary	88	49			
	Others	1	1			
Level of education (Females)	Primary	64	4	27.153	2	*p* = 0.000
	Secondary	141	53			
	Tertiary	55	41			
Occupation (Males)	Formal employment	73	48	19.7541	4	*p* = 0.0056
	Casual labourer	34	15			
	Self employed	121	29			
	Unemployed	32	4			
	In school	1	1			
Occupation (Females)	Formal employment	48	20	15.9208	4	*p* = 0.003
	Casual labourer	20	19			
	Self employed	109	33			
	Unemployed	50	37			
	In school	12	10			
Place of residence (Male)	Rural	144	29	19.009	2	*p* = 0.000
	Urban	79	46			
	Peri urban	37	23			
Place of residence (Female)	Rural	153	31	21.8155	2	*p* = 0.000
	Urban	64	44			
	Peri urban	43	23			

*df*, degrees of freedom.

#### Waiting time for cervical cancer screening services

Majority of the participating couples did not like waiting for more than an hour for the cervical screening services. Using Chi-square test and a significance level of *p* ≤ 0.05, there was statistical significance in the interaction between waiting time and the following socio-demographic and economic variables, *p* = 0.04 for females age, *p* = 0.0036 for the level of education for males, *p* = 0.001 for female occupation and *p* = 0.0068 for the main income earner.

#### Distance to the clinic offering cervical cancer screening services

More than 60.0% of participants preferred the clinic or hospital offering cervical cancer screening services to be less than one km for the male partners to actively participate. There was some statistical significance in the interaction between the clinic distance and some of the socio-demographic and economic characteristics when using Chi-square test at significance level of *p* ≤ 0.05 – age of male partner *p* = 0.01, marital status *p* = 0.001, male level of education *p* = 0.01, female level of education *p* = 0.01 and female occupation *p* = 0.000.

#### Cost of cervical cancer screening services

Health facility costs interaction with marital status (*p* = 0.0001, *χ*^2^ = 37.896), level of education for males (*p* = 0.0001, *χ*^2^ = 63.610), females’ level of education (*p* = 0.0001, *χ*^2^ = 31.715), male occupation (*p* = 0.000, *χ*^2^ = 52.24), female occupation (*p* = 0.0000, *χ*^2^ = 342.25), main income earner (*p* = 0.001, *χ*^2^ = 31.76), place of residence for male (*p* = 0.001, *χ*^2^ = 48.158) and place of residence for females (*p* = 0.001, *χ*^2^ = 46.606), was significant at *p* ≤ 0.05. Across the majority of the socio-demographic characteristics, the study participants preferred health facility cost of ≤ 100 Ksh.

### Evaluation of the association between male partner socio-demographic characteristic, health facilities and community attributes and utilisation of human papillomavirus vaccination services

As illustrated in Table 6, at confidence level of 95% (significance level of ≤ 0.05), marital status (*p* = 0.017, *χ*^2^ = 10.19), occupation (*p* = 0.001, *χ*^2^ = 49.603), main income earner (*p* = 0.0438, *χ*^2^ = 6.2552), place of residence (*p* = 0.0000, *χ*^2^ = 26.74), friendly affordable cost (*p* = 0.025, *χ*^2^ = 9.357), community members support (*p* = 0.001, *χ*^2^ = 43.449), places of service delivery (*p* = 0.001, *χ*^2^ = 17.89) and male-friendly services (*p* = 0.0000, *χ*^2^ = 37.6) had statistical significance.

**TABLE 6:** Male socio-demographic and economic characteristics, health facility attributes and utilisation of human papillomavirus vaccination services.

Variables		Will you take your children for HPV vaccination?		*χ*^2^ value	*df* value	*p*-value
Attributes	Characteristics	Yes	No			
Marital status	Cohabiting	94	21	10.19	3	*p* = 0.017
	Customarily marriage	138	57			
	Civil marriage	41	5			
	Others	1	1			
Occupation (Male)	Formal employment	115	6	49.063	4	*p* < 0.001
	Casual labourer	24	25			
	Self-employed	104	46			
	Unemployed	29	7			
	In school	2	0			
Main income earner	Female	75	14	6.2552	2	*p* = 0.0438
	Male	148	58			
	Both	48	11			
Place of residence (Male)	Rural	115	58	26.74	2	*p* = 0.000
	Urban	115	10			
	Peri urban	44	16			
Friendly affordable cost	Free of charge	24	0	9.357	3	*p* = 0.025
	Less than 100 Ksh	142	42			
	100 Ksh	92	37			
	More than 100 Ksh	16	5			
Community members support	Teachers	8	1	43.449	4	*p* < 0.001
	Church elders	17	2			
	Peers/friends	23	19			
	Community health workers	151	17			
	Community leaders	75	45			
Places for service delivery	Clinic setup	133	23	17.89	2	*p* < 0.001
	Institution like school setup	11	11			
	Community outreach setups	129	50			
Types of male-friendly services	More male health workers	48	65	37.6	4	*p* = 0.000
	Gender-sensitive sitting arrangements	1	38			
	Health education focusing on male partners	14	69			
	Other male partner-oriented services	14	77			
	Treatment of STIs	7	25			

HPV, human papillomavirus; *df*, degrees of freedom; Ksh, Kenyan shillings; STIs, sexually transmitted infections.

#### Relationship between female socio-demographic and economic characteristics and the intention to involve her male partner during cervical cancer screening processes

A linear regression analysis was conducted to evaluate the extent to which female partners socio-demographic and economic characteristics could predict their intention to involve their male counterparts during the process of cervical cancer screening.

As shown in Table 7, a significant regression was found (*F* [3354] = 7.843, *p* = < 0.000). The *R*^2^ was 0.062, indicating that female partner socio-demographic and economic characteristics explained approximately 6.2% of the variance in their intention to involve their male partners.

**TABLE 7a:** Linear regression on female socio-demographic factors and intention to involve her male partner.

Model summary				
Model	*R*	*R* square	Adjusted *R* square	s.e. of the estimate
1	0.250^a^	0.062	0.054	0.380

s.e., standard error.

a Predictors: (constant), What is your occupation (female), What is your education level (female), Age of female in years.

**TABLE 7b:** Linear regression on female socio-demographic factors and intention to involve her male partner.

Analysis of variance (ANOVA^a^)					
Model 1	Sum of squares	*df*	Mean square	*F*	Sig.
Regression	3.394	3	1.131	7.843	0.000^b^
Residual	51.067	354	0.144	-	-

**Total**	**54.461**	**357**	**-**	**-**	**-**

*df*, degrees of freedom; Sig., significance.

a Dependent variable: Do you intend to involve your partner when screening for cervical cancer.

b Predictors: (constant), What is your occupation (female), What is your education level (female), Age of female in years.

**TABLE 7c:** Linear regression on female socio-demographic factors and intention to involve her male partner.

Coefficients^a^					
Model 1	Unstandardised coefficients		Standardised coefficients	*t*	Sig.
	*β*	s.e.	*β*		
(Constant)	=0.850	0.156	-	5.459	0.000
Age of female in years (constant)	−0.004	0.003	−0.081	−1.407	0.160
What is your education level (female)	0.097	0.033	0.168	2.993	0.003
What is your occupation (female)	−0.034	0.013	−0.144	−2.685	0.008

s.e., standard error; Sig., significance; *β*, Beta.

a Dependent variable: Do you intend to involve your partner when screening for cervical cancer?

## Discussion

The study focussed on a quantitative approach to couples’ perception on the roles, patterns and factors associated with male partner involvement in transmission, prevention and control of cervical cancer. This is based on the premise that cervical cancer should be viewed not just as a health issue affecting individual women but also as a social issue that requires greater public awareness of the male role in both transmission and prevention (Moodley & De Vries 2016). Engaging men through advocacy, education and involvement in cervical cancer screening within socio-cultural contexts is crucial, as it enables them to make informed decisions regarding their partner’s screening. Establishing how male partners can be involved or why they are not involved during cervical cancer prevention and control measures is important as in many African settings, men continue to hold superior positions within the family (Adegboyega et al. 2019).

### Extent of male partner involvement in cervical cancer prevention and control

The study found that male partner involvement in cervical cancer screening is influenced by various factors. Three main areas for improving male participation in cervical cancer prevention and control were identified: (1) individual male characteristics, which are largely shaped by their environment; (2) the healthcare system, including the quality and delivery of care, health service providers and health facilities and (3) community engagement and leadership. Clear guidelines are needed to effectively mobilise males for participation in these processes.

#### Socio-demographic and economic characteristics

The study revealed that various socio-demographic factors influence men’s involvement in their partner’s cervical cancer screening. Marital status emerged as a significant factor, with formally married men showing greater engagement, including a willingness to take their children for HPV vaccination. This observation is consistent with a study done in Malawi, which found that stably married men were more likely to have a positive attitude towards gender equity in sexual matters and were more actively involved in their partner’s cancer screening (Lewis et al. 2020). In addition, the male partner’s area of residence was linked to their involvement in HPV vaccination services. The physical environment and social determinants of health impact healthcare behaviours (Artiga & Hinton 2018).

Socio-demographic and economic factors considered in this study included: income, occupation and education (see Table 1). Education-wise, 38.0% (*n* = 137) had tertiary education, which can contribute to high awareness of cervical cancer. Education level was associated with men’s involvement in cervical cancer screening (Table 5). This finding is consistent with a Swedish study that indicated that education levels were associated with uptake of cervical cancer screening services (Broberg et al. 2018). The findings is also in concurrence with research done in Iran, which demonstrated that poor reproductive health was less common among those with higher levels of education (Khazaeian et al. 2018). Poor education leads to inadequate knowledge, which could contribute to a low level of male involvement in their partner(s) cervical cancer screening. This study also reported that only 33.8% (*n* = 121) of male respondents were formally employed, and the least percentage was either self-employed, casual labourers or unemployed (Table 1). There was a strong association (*p* = 0.000) on male occupation, main income earner (*p* = 0.04) and the propensity of male participating in cervical cancer prevention services (see Table 6). This indicates that males in the study area could not effectively support their partners on matters related to cervical cancer screening; hence, financial factors continue to hinder the uptake of screening services. These findings are in agreement with a study’s report from Nepal and from South West Nigeria, which concluded that the lack of money is one of the obstacles to the screening of cervical cancer and that cervical cancer screening is influenced by economic or financial barriers (Darj, Chalise & Shakya 2019; Onyenwenyi & Mchunu 2018). These challenges include financial constraints such as the cost of travel to distant screening clinics and hospitals. These could have influenced men’s involvement in cervical cancer screening procedures of their female partners.

The main income earner among the couples (Table 1) was the male partner (57.5%, *n* = 206). This implies that men were more empowered economically than their partners, and this could affect decision making regarding health care seeking behaviour. If the male partner does not support the spouse financially, it may lead to non-utilisation of the availed cervical cancer prevention health services. This concurs with findings from a study done on follow up after an abnormal cervical cancer screening result and the role of male partners in Malawi, where the researchers identified the lack of financial support (especially for transport) from the male partner as a barrier in presenting for follow-up care (Chapola et al. 2021).

#### Health care system attributes

To ensure sustainability of male involvement in cervical cancer screening services, as shown in Table 2, majority of the couples were okay with cervical cancer screening clinics opening on week days (72.6%, *n* = 260), 26.5% (*n* = 95) preferred them being open even during weekends while less than 1% (*n* = 3) were okay with either. Majority of the couple participants wanted the waiting time to be less than 1 h (83.2%, *n* = 298) while 16.8% (*n* = 60) were not bothered by waiting for an hour or more. Majority of the couples preferred the hospital to be less than one km from their homesteads (68.2%, *n* = 244), 25.7% (*n* = 92) wanted it to be at least one km while 6.1% (*n* = 22) were okay even when the hospital providing cancer of the cervix screening services was more than one km.

Majority of the couples preferred the hospital charge to be less than 100 Ksh (51.4% = 184), 36.0% (*n* = 129) wanted it to be at least 100 Ksh and 5.9% (*n* = 21) were okay with more than 100 Ksh. Noteworthy is that only 6.7% (*n* = 24) wanted the screening services to be free (Table 2).

The findings from this study show that the health facilities need to: be accessible geographically (less distance) and financially (less fare to the facility), have convenient opening time and less waiting time and have a friendly and affordable service fee in order to ensure sustainability of male partner attendance. These findings are in agreement with those reported in a systematic review study entitled ‘Underutilization of cervical cancer prevention services in low- and middle-income countries’, which concluded that for screening services to be acceptable, they should be offered at the right time, in the right place, at the right fee and in the right manner (Chidyaonga-Maseko et al. 2015). In yet another study on ‘Environmental and psychosocial barriers to and benefits of cervical cancer screening in Kenya’, challenges to cervical cancer screening identified were poor availability of services, long travel distances to screening locations, extended waiting hours and high screening costs (Buchanan Lunsford et al. 2017).

The most preferred location to offer male-friendly cervical cancer screening services was reported by the participant couples to be at the community outreach level (50%, *n* = 179) as shown in Table 6. The community-based approaches as advocated for by the participants could be necessitated by the need for convenience in terms of accessibility as most clients spend their time within the community and therefore may not need the fare to travel to clinics or hospitals. The national cancer screening guidelines of 2018 recommend models of cancer screening service provision, each with an essential service package (MOH 2018). What should be experimented therefore is decentralisation of the clinic-based model to a community-based model. The findings of this study concur with the study ‘Design and evaluation of a theory-based, culturally relevant outreach model for breast and cervical cancer screening for Latina immigrants’, which reported that using a theoretical approach to outreach design and implementation, was possible to reach more Latina immigrants and connect them to cancer screening services (White et al. 2012).

The findings of this study also largely indicated service provider’s attributes and behaviour are key to sustainability of male partner involvement in cervical cancer prevention and control processes. The health service provider’s attributes included: being non-judgemental, polite, welcoming and maintaining confidentiality. These findings are substantiated by a study done in sub-Saharan Africa that reported negative health service providers attitude, poor knowledge and skills of youth and the lack of essential drugs and equipment as the factors associated with inadequate provision of the SRH services (Jonas et al. 2017). Other studies concurring with these findings include systematic review on barriers and facilitators to uptake of cervical cancer screening among Ugandan women (Black, Hyslop & Richmond 2019) and another one on underutilisation of cervical cancer prevention services in low- and middle-income countries (Chidyaonga-Maseko et al. 2015). Both these studies reported that discourtesy among health care providers discourages clients from utilising the availed health care services.

The type of male-friendly services the couples preferred during the cervical cancer prevention and control health services included more male health care providers (31.6%, *n* = 113), male partner-oriented services such as prostate cancer screening (25.4%, *n* = 91), health education focussing on male partners (23.2%, *n* = 83), gender-sensitive sitting arrangements (10.9%, *n* = 39) and treatment of sexually transmitted infections (8. 9%, *n* = 32) as depicted in Table 6. From male perspective, for them to be attending the screening clinics together with their partners, more male health service providers were needed. This was in contrast with the findings from the study done in Malawi on exploring barriers to the delivery of cervical cancer screening and treatment where it was reported that the delivery of cervical cancer screening and early treatment services was compromised because of factors such as acute shortage of staff, the lack of equipment and supplies, the lack of supportive supervision and the use of male service providers (Munthali, Ngwira & Taulo 2015). Yet, in another study on factors influencing male participation in reproductive health in Nepal, the findings showed limited male involvement with participants reported several hindering and challenging factors such as sociocultural and psychological norms, the lack of education and misinformation and dominance of female as health care providers in many MCH clinics (Sharma et al. 2018).

#### Family and community support attributes

Majority of the couples felt that male partners can be involved in the cervical cancer prevention and control processes through moral encouragement to go for the checkups (26.8%, *n* = 96), provide resources such as funds (25.1%, *n* = 90), accompany the female partner to the clinic (19.0%, *n* = 68), give advice (12.6%, *n* = 45), be less judgemental (12.6%, *n* = 45) and avoid discrimination and stigmatisation (3. 6%, *n* = 13) as shown in Table 3. The findings correspond with a study done in Western Kenya on female’s perspective on male involvement in HPV-based cervical cancer screening where it was noted that women experienced both support and opposition from their male partners. The support was in the form of financial support for transportation, emotional support and encouragement, while opposition ranged from anticipated negative reactions to the lack of permission, isolation and abandonment (Adewumi et al. 2019). Another study that supports the findings was on male support for cervical cancer in rural Ghana that found out men provided various forms of support – financial, social, material and emotional – to their partners during the screening and treatment stages of the disease. Some men, however, abandoned their partners during the screening and treatment process of the disease (Binka et al. 2019).

While 76.5% (*n* = 274) of the couples were willing to take their children for HPV vaccines, 23.5% (*n* = 84) were not willing. Some of the reasons for not using the HPV vaccination services were unawareness of existence of such services (18.4%, *n* = 66), the lack of trust of the HPV vaccines (3.9%, *n* = 14) and unawareness of where the vaccination services are offered (1.1%, *n* = 4).

Majority of the respondents, 73.7% (*n* = 264), were not aware that HPV is a sexually transmitted infection, which illustrated how male partners who had multiple sexual partners were not even aware that they were risking their partners to cervical cancer.

Some of the community members the couples felt could assist in encouraging male partners to support their women to screen for cervical cancer included community health workers (46.9%. *n* = 168), community leaders (33.5%, *n* = 120), peers or friends (11.7%, *n* = 42) and teachers (2.5% *n* = 9) as shown in Table 6. When women were asked independently and confidentially about their intention to involve their male partners during the cervical cancer screening processes, majority (81.3%, *n* = 291) were willing to involve their men, while 18.7% (*n* = 67) were not willing.

The reasons for involving their male partner included financial support (47.9%, *n* = 171), moral support (22.1%, *n* = 79), to understand the process (5.7%, *n* = 21), out of love for them (3.1%, *n* = 11), it is their marital duty (1.9%, *n* = 7), while 0.6% (*n* = 2) did not know why.

Some of the reasons for not involving their male partner in the cervical cancer screening processes included; being busy that is not being available (6.7%, *n* = 24), not concerned or supportive about the screening services (4.7%, *n* = 17), women liking their privacy and confidentiality (3.1%, *n* = 11), their men being judgemental (2.2%, *n* = 8), their men being shy (1.4%, *n* = 5), while 0.6% (*n* = 2) did not have a reason (see Table 4).

Findings from the study show that women or female partners desire their male partners to be part of the process when they are being screened and treated not only through financial support but also morally and emotionally. This makes the whole process bearable psychologically, and it might even make the couples bond strong. With the right exposure and proper health education, men are motivated and willing to assist their partners in the processes of cervical cancer prevention and control. This is supported by findings from the study on ‘Involving men in cervical cancer prevention: A qualitative enquiry into male perspectives on screening and HPV vaccination in Mid-Western Uganda’, where Ugandan men were willing to support cervical cancer prevention for their wives and daughters after being informed about cervical cancer (De Fouw et al. 2023).

### Evaluation of the association between variables influencing male participation in cervical cancer prevention and control

The interaction between convenient working days and socio-demographic and economic characteristics showed statistical significance at the significance level of *p* ≤ 0.05 with *p* = 0.003 for males age, *p* = 0.0226 for females age, *p* = 0.038 for marital status, *p* = 0.0004 for the level of education for males, *p* = 0.000 for female’s level of education, *p* = 0.003 for female occupation and *p* = 0.000 for the place of residence for both males and females (see Table 5). The findings imply that there are some socio-demographic attributes that will affect the individual perception of the health system factors and then influence his uptake of a particular health care service. For instance, female level of education will affect the days she perceives to be conducive for her attendance to screening because of her official busy schedule. Also, marital status may determine when the woman will go to the clinic and if to be accompanied by her spouse.

Majority of the participating couples did not like waiting for more than an hour for the cervical screening services. Using Chi-square test and a significance level of *p* ≤ 0.05, there was statistical significance in the interaction between waiting time and the following socio-demographic and economic characteristics: *p* = 0.04 for females age, *p* = 0.0036 for the level of education for males, *p* = 0.001 for female occupation and *p* = 0.0068 for the main income earner.

All these socio-demographic and economic factors will determine an individual level of patience to access the health service. In case a male partner experiences delay at the health services delivery point when he had volunteered to take his spouse, the motivation of coming again is diminished.

More than 60.0% (*n* = 249) of participants preferred the clinic or hospital offering cervical cancer screening services to be less than one km for the male partners to actively participate. There was some statistical significance in the interaction between the clinic distance and some of the socio-demographic and economic characteristics such as age of male partner *p* = 0.01, marital status *p* = 0.001, male level of education *p* = 0.01, female level of education *p* = 0.01 and female occupation *p* = 0.000. The issue of proximity to the health centre is an important enhancer or deterrence, but other factors such as the level of education of both partners and whether they are married may play a role in deciding to utilise these services and also support each other.

Health facility cost interaction with marital status (*p* = 0.0001, *χ*^2^ = 37.896), level of education for males (*p* = 0.0001, *χ*^2^ = 63.610), females’ level of education (*p* = 0.0001, *χ*^2^ = 31.715), male occupation (*p* = 0.000, *χ*^2^ = 52.24), female occupation (*p* = 0.0000, *χ*^2^ = 342.25), main income earner (*p* = 0.001, *χ*^2^ = 31.76), place of residence for males (*p* = 0.001, *χ*^2^ = 48.158) and place of residence for females (*p* = 0.001, *χ*^2^ = 46.606) were significant at *p* ≤ 0.05. Across majority of the socio-demographic factors, the study participants preferred health facility cost of ≤ 100 Ksh. The cost of health care services contributes a lot in uptake of the health services offered. This is further influenced by other socio-demographic and economic factors of the individuals or families.

The study found a significant association between socio-demographic and economic characteristics of the male partner, health facilities, community attributes and utilisation of HPV vaccination services: marital status (*p* = 0.017), occupation (*p* = 0.001), main income earner (*p* = 0.0438), place of residence (*p* = 0.000), friendly affordable cost (*p* = 0.025), community members support (*p* = 0.001), places of service delivery (*p* = 0.001), male-friendly services (*p* = 0.000). This indicates that when planning for campaigns to involve males to participate in programmes or activities related to cervical cancer prevention and control, it is important to consider their marital status, occupation, economic activities and the cost of the screening services. The services should also be male friendly and have community members including gatekeepers support. This is consistent with the study done in Zimbabwe on women and health providers’ perspectives on male support for cervical cancer screening. Involvement of community leaders was seen as crucial in the facilitation of male involvement for programme acceptance and improved uptake of cervical cancer screening (Mantula & Toefy 2023).

A linear regression analysis was conducted to evaluate the extent to which female partners’ socio-demographic and economic characteristics could predict their intention to involve their male counterparts during the process of cervical cancer screening as shown in Table 7.

A significant regression was found (*F* [3354] = 7.843, *p* = < 0.000), where female partner’s socio-demographic and economic characteristics explained approximately 6.2% of the variance in their intention to involve their male partners.

The finding concurs with the study done on female perspectives on male involvement in a HPV-based cervical cancer-screening programme in western Kenya, where the majority of women described their own partners as supportive based on their personal characteristics but many felt that other male partners would not be supportive. Most women believed that increased HPV and cervical cancer awareness and knowledge would increase partner support. Participants reported a general acceptance of involvement of community leaders in education and screening campaigns, in a setting where such leaders may hold influence over males in their locality (Adewumi et al. 2019).

## Recommendations

Based on study findings, policymakers can develop and implement national policies that integrate male partner involvement into existing national health programmes and campaigns, ensuring that messages and strategies are tailored to reach men effectively. There should be focus on promoting awareness and education among men about cervical cancer and the importance of their role in prevention, including HPV vaccination and regular cervical cancer screening for their partners. Researchers can conduct further research to explore the effectiveness of different interventions and strategies for promoting male partner involvement in cervical cancer prevention and control.

## Conclusion

Couples in the study agreed that male partner’s individual constructs play an important role in cervical cancer transmission and prevention. This includes socio-demographic and economic characteristics and communication patterns with partners. The health care system, including the infrastructure layout, distance to where services are offered, type of services offered and health workers’ reception, will influence male partner’s participation in cervical cancer prevention. The male partner’s interaction with the environment that includes their immediate families, community members and leaders affects their involvement in the processes of cervical cancer prevention and control. The study also found that there was less awareness among couples about transmission, prevention and control of cervical cancer especially on HPV.
